# Clinical Features Associated with Frozen Shoulder Syndrome in Parkinson's Disease

**DOI:** 10.1155/2015/232958

**Published:** 2015-06-09

**Authors:** Ya-Ting Chang, Wen-Neng Chang, Nai-Wen Tsai, Kuei-Yueh Cheng, Chih-Cheng Huang, Chia-Te Kung, Yu-Jih Su, Wei-Che Lin, Ben-Chung Cheng, Chih-Min Su, Yi-Fang Chiang, Cheng-Hsien Lu

**Affiliations:** ^1^Department of Biological Science, National Sun Yat-sen University, Kaohsiung, Taiwan; ^2^Department of Neurology, Chang Gung Memorial Hospital, Kaohsiung Medical Center, Chang Gung University, College of Medicine, Kaohsiung 833, Taiwan; ^3^Department of Emergency Medicine, Chang Gung Memorial Hospital, Kaohsiung Medical Center, Chang Gung University, College of Medicine, Kaohsiung 833, Taiwan; ^4^Department of Medicine, Chang Gung Memorial Hospital, Kaohsiung Medical Center, Chang Gung University, College of Medicine, Kaohsiung 833, Taiwan; ^5^Department of Radiology, Chang Gung Memorial Hospital, Kaohsiung Medical Center, Chang Gung University, College of Medicine, Kaohsiung 833, Taiwan; ^6^Department of Neurology, Xiamen Chang Gung Memorial Hospital, Xiamen, Fujian, China

## Abstract

*Background*. Frozen shoulder syndrome is a common musculoskeletal disease of idiopathic Parkinson's disease (PD) that causes long-term pain and physical disability. A better understanding of the associated factors can help identify PD patients who will require prevention to improve their quality of life. *Methodology*. This prospective study evaluated 60 shoulders of 30 PD patients. Correlation analysis was used to evaluate the relationships between clinical factors and shoulder sonography findings. *Results*. Frozen shoulder syndrome was found in 14 of 30 PD patients affecting 19 shoulders, including bilateral involvement in five and unilateral involvement in nine. There was a significant positive correlation between the parameters of sonography findings and frozen shoulder syndrome (i.e., thickness of bicipital effusion and tendon thickness of the subscapularis and supraspinatus) and mean ipsilateral Unified Parkinson's Disease Rating Scale (UPDRS) III and its subscores (tremor, rigidity, and bradykinesia scores). *Conclusions*. Higher ipsilateral UPDRS and subscores are associated with increased effusion around the biceps tendon, with increased tendon thickness of subscapularis and supraspinatus. Preventing frozen shoulder syndrome in the high-risk PD group is an important safety issue and highly relevant for their quality of life.

## 1. Introduction

After Alzheimer's disease, Parkinson's disease (PD) is the second most common neurodegenerative disorder [1–4]. Associated musculoskeletal conditions, seen in as high as 70% of PD patients, are the most common cause of long-term pain and physical disability [5]. Frozen shoulder syndrome, also known as adhesive capsulitis, is characterized by stiffness and pain and is associated with increased fluid in the tendon sheath of the long head of the biceps [6]. It is one of its most disabling musculoskeletal problems severely affecting the quality of life of patients with PD.

To date, very few clinical researches have focused specifically on PD patients with frozen shoulder syndrome [7–10]. Most of these studies are case reports [11] or retrospective studies [7] with less strict selection criteria (e.g., enrolled in combination with PD and multiple system atrophy or frozen shoulder and other shoulder disturbances) [12, 13] or depend solely on clinical diagnosis [14].

Sonography enables the visualization of the internal structure of the shoulder tendons and has become an important diagnostic adjunct in the evaluation of shoulder conditions [15, 16]. Although sonography and magnetic resonance imaging have comparable high sensitivity and specificity for diagnosing rotator pathologies [15, 16], ultrasonography is noninvasive, more widely accessible, portable, and less expensive for assessing soft tissue involvement in musculoskeletal diseases [17].

Because of its possible benefits, an appropriate rehabilitation program should be routinely recommended, with emphasis on regular physical activity to reduce the risk of frozen shoulder syndrome. Nonetheless, a better delineation of potential prognostic factors in PD patients who should receive preventive intervention is warranted. At the same time, it is imperative to obtain accurate information on the relative frequency and severity of frozen shoulder syndrome, the severity and duration of PD, the daily dose of antiparkinsonian agents, and functional outcome. Thus, this hospital-based study aimed to analyze the clinical features, imaging findings, scientific clinical scores, and measurements to determine potential risk factors associated with frozen shoulder syndrome in PD patients.

## 2. Patients and Methods

### 2.1. Study Design

This single-center prospective, case-control study was conducted at Chang Gung Memorial Hospital, Kaohsiung, a tertiary medical center and the main referral hospital serving a population of 3 million in southern Taiwan.

### 2.2. Inclusion Criteria

This study evaluated 60 shoulders of 30 patients with clinical diagnosis of idiopathic Parkinson's disease with a good levodopa response [18], who were followed up at the Neurology Out-Patient Clinic for more than six months after titration of their daily antiparkinsonian agents to a steady dose in accordance with their clinical symptoms. There were 34 patients initially enrolled in this study. Four patients including rotator cuff tear in 2 and shoulder joints dislocation in 2 were excluded. Finally, only 30 cases were enrolled in this study. Seventeen shoulders of nine healthy age-matched patients who did not have PD were studied as the control group. All of the subjects in the control group were asymptomatic for shoulder pathologies and were pain-free.

The hospital's Institutional Review Committees on Human Research approved the study protocol and all of the patients or their relatives provided written informed consent.

### 2.3. Exclusion Criteria

Patients with preexisting shoulder pathology or other medical history of stroke, rheumatoid arthritis, trauma, dislocation, fracture or surgery, or cervical spine disease were excluded.

### 2.4. Clinical Assessment

An experienced neurology nurse specialist who was blinded to the patients' clinical and biochemical data was trained to measure the functional scores upon enrollment. The clinical features recorded were age at disease onset (or age at the time of the first reported symptom attributable to the disease) and disease duration (time from onset until follow-up). The severity of PD was graded according to the scores of the Unified Parkinson's Disease Rating Scale (UPDRS) III and the Hoehn and Yahr staging [19, 20].

The daily dose of antiparkinsonian agents was converted into the equivalent dose of levodopa [21]. In patients with fluctuating PD, the UPDRS III and Hoehn and Yahr scales were administered during the “off” situation (8–10 hours after the patient stopped the usual antiparkinsonian treatment) to evaluate the possible influence of disease severity. The Mini-Mental State Examination (MMSE) was used to assess general intellectual function, while cognitive outcomes were assessed using the Clinical Dementia Rating (CDR) scale. All of the subjects were assigned a CDR rating score, as follows: 0 for no dementia and 0.5, 1, 2, and 3 for questionable, mild, moderate, and severe dementia, respectively.

All of the patients underwent ^99m^Tc-TRODAT-1 SPECT/CT study according to previously published methods [22]. The Hoehn and Yahr disability scale and UPDRS III were used to determine the severity of the disease. Both shoulders were used separately for data analysis.

### 2.5. Diagnostic Criteria of Adhesive Capsulitis

In this study, the diagnostic criteria for adhesive capsulitis were the following [23]: (1) insidious onset of pain associated with passive glenohumeral motion; (2) restricted range of glenohumeral motion both actively and passively, with external rotation <50% of the normal side; and (3) normal radiography and a shoulder ultrasound demonstrating no significant rotator cuff tear. Patients were excluded if they had secondary causes of adhesive capsulitis such as a fracture, recent shoulder surgery, calcific tendonitis, or rotator cuff tears; declined to participate; or were younger than 18 years of age. Four patients were excluded for shoulder dislocation in one, rotator cuff tear in two, and another shoulder disorder in one. Only 30 cases were finally included in this study.

### 2.6. ^99m^Tc-TRODAT-SPECT/CT and Region of Interest Analysis

The participants were intravenously injected with a single bolus dose of  ^99m^Tc-TRODAT-1([2[[2-[[[3-(4-chlorophenyl)-8-methyl-8-azabicyclo[3,2,1]-oct-2-yl]-methyl](2-mercaptoethyl)amino]ethyl]amino]ethanethiolato(3-)-N2,N2′,S2,S2]oxo-[1R-(exo-exo)]-^99m^Tc-technetium). Four hours after the injection, images were obtained using a dual-head SPECT/CT equipped with low-energy high-resolution collimators (Symbia T, Siemens Medical Solutions, Erlangen, Germany). With the help of anatomic coregistration CT images, the regions of interest (ROIs) of the bilateral striata (including their subregions of caudate and putamen) were defined on composite images of the six highest striatum activity slices. The occipital cortex was drawn in the same way and served as background area. The radioactivity of the regions of interest was then calculated.

Striatal dopamine transporter uptake ratios (TRODAT ratio) were calculated as the quotient of the mean counts per pixel in each striatum divided by the mean counts per pixel in the occipital cortex. All images were reviewed by an experienced nuclear physician who was blinded to the patients' data.

### 2.7. Sonographic Examination

An experienced neurologist who was skilled in shoulder sonography examinations and blinded to the patients' clinical and biochemical data was trained to measure shoulder sonography studies at the time of enrollment. Ultrasonography of the shoulder was performed using a scanner with a 12/5 MHz linear array transducer (Philips HDI 5000; Philips Medical Systems, Bothell, WA). During the examination, the patient sat on a stool in a comfortable position and the long head of the biceps was examined with the patient's hands placed on the ipsilateral knee. The subscapularis tendon and biceps tendon grooves were examined with the patient seated and the arm held in neutral position, the elbow flexed to 90°, and the forearm resting supine on the ipsilateral thigh, dynamic with internal and external rotation [24–27]. The supraspinatus tendon was evaluated in full internal rotation and hyperextension, with the forearm behind the back and the palm facing posteriorly [24–27]. The infraspinatus tendon was evaluated with the bent arm placed in front of the patient's own chest, while the ipsilateral hand was on the contralateral arm [24–27].

All of the tendons were examined in transverse and longitudinal scans, and, after adjusting the angle of measurement, the brightest echogenic tendon was obtained. Tendon thickness was measured in sonograms at the point of the largest diameter. An increased hypoechoic area (thickness > 2 mm) around the long head of the biceps tendon in the transverse view was interpreted as effusion in the biceps tendon sheath [27]. Nonvisualization of the tendon or complete fiber discontinuity of the subscapularis, supraspinatus, and infraspinatus tendons was interpreted as full thickness tear [25]. Partial fiber discontinuity and mixed hypo- and hyperechoic focus in the critical zone of the tendon suggested partial thickness tear [26]. Tendonitis was defined as hypoechoic and swelling changes, with a difference in tendon thickness (>2 mm) compared to the healthy side [25].

### 2.8. Statistical Analyses

Data were expressed as mean ± SD or median (interquartile range) for continuous variables. Those with skewed deviation were logarithmically transformed to improve normality before comparison. Two separate statistical analyses were performed. First, parameters of sonography findings among three groups were compared using one-way ANOVA, followed by post hoc multiple comparison. Comparisons between two groups were made using independent *t*-test. Second, correlation analysis was used to explore the relationship between parameters of shoulder sonography findings and clinical severity in PD patients. All statistical analyses were conducted using the SPSS software package, version 12 (SPSS, Inc., Chicago, IL).

## 3. Results

The 30 patients with idiopathic PD included 14 males (age range, 47–78 years; mean age, 66.6 years) and 16 females (age range, 47–81 years; mean age, 63.2 years) (Table 1). Frozen shoulder syndrome was found in 19 shoulders of 14 patients, including bilateral involvement in five and unilateral involvement in nine. Furthermore, the mean age between patients with and without frozen shoulder was 64.9 ± 10.9 and 64.7 ± 10.2, respectively (*p* = 0.965), and the mean educational levels between patients with and without frozen shoulder were 6.4 ± 3.1 and 4.6 ± 3.2, respectively (*p* = 0.130).

The underlying diseases of the 30 patients included hypertension in 12, diabetes mellitus (DM) in six, and knee osteoarthritis in four (Table 1). Their percentage of cognitive impairment was 46.6%, including CDR 0.5 in 13 and CDR 1 in one. By the Hoehn and Yahr staging, 11 were of stage I, nine were of stage II, eight were of stage III, and two were of stage IV. Their mean UPDRS III score was 27.73 ± 13.9 (range, 5–62).

The mean thickness of the tendon in the 19 shoulders with adhesive capsulitis was as follows: subscapularis 5.10 ± 1.2 mm, supraspinatus 5.45 ± 1.2 mm, and infraspinatus 5.52 ± 1.3 mm. The mean thickness of the bicipital effusion in the 19 frozen shoulders was 2.72 ± 0.5 mm. The ipsilateral UPDRS of PD patients with and without frozen shoulder syndrome was 17.00 ± 7.1 and 14.07 ± 8.8 (*p* = 0.210), respectively, while their other ipsilateral motor severities were listed in Table 2. Patients with frozen shoulder had a mean MMSE of 25.43 ± 3.3 points, whereas patients without frozen shoulder had a mean MMSE of 23.81 ± 7.7 points.

Correlation analysis was used to test the influence of MMSE and ipsilateral motor state (ipsilateral UPDRS III, ipsilateral tremor score, ipsilateral rigidity score, and ipsilateral bradykinesia score) on mean effusion around the biceps tendon. The significant statistical results (correlation coefficient,* p* value) revealed a mean MMSE score (*r* = −0.341, *p* = 0.035), mean ipsilateral UPDRS III score (*r* = 0.618, *p* < 0.001), mean ipsilateral tremor score (*r* = 0.521, *p* < 0.001), mean ipsilateral rigidity score (*r* = 0.574, *p* < 0.001), and mean ipsilateral bradykinesia score (*r* = 0.613, *p* < 0.001).

Furthermore, the mean ipsilateral UPDRS III score, mean ipsilateral tremor score, mean ipsilateral rigidity score, and mean ipsilateral bradykinesia score were positively correlated with the thickness of the subscapularis tendon (average) (*r* = 0.386, *p* = 0.003; *r* = 0.465, *p* < 0.001; *r* = 0.281, *p* = 0.031; and *r* = 0.399, *p* = 0.002, resp.), while the mean ipsilateral UPDRS III score (*r* = 0.421, *p* = 0.001), mean ipsilateral tremor score (*r* = 0.341, *p* = 0.004), and mean ipsilateral bradykinesia score (*r* = 0.385, *p* = 0.001) were positively correlated with the thickness of the supraspinatus tendon (average) (Table 3).

## 4. Discussion

Differences in the relative prevalence of frozen shoulder syndrome in patients with idiopathic PD vary with case determination and inclusion criteria, disease severity, length of follow-up, underlying diseases, and diagnostic methods. The frequency of frozen shoulder syndrome in PD patients in other studies varies and is estimated to be as high as 22.6% in one study [7]. In the present study, the frequency is 46.7% (14 out of 30).

The present study examines risk factors associated with frozen shoulder syndrome in 30 patients with idiopathic PD and has two major findings. First, both the mean thickness of the subscapularis tendon and the supraspinatus and infraspinatus muscles and the thickness of bicipital effusion in the shoulders are largest in the frozen shoulders of PD patients, followed by the nonfrozen shoulders of PD patients and the shoulders of healthy controls. Second, both the mean thickness of the subscapularis and supraspinatus tendons and the thickness of bicipital effusion in the shoulders positively correlated with the mean ipsilateral UPDRS III and its subscores (i.e., tremor, rigidity, and bradykinesia scores).

Frozen shoulder syndrome, also known as adhesive capsulitis, is the spontaneous onset of painful and progressively severe restriction of shoulder joint mobility, in the absence of any demonstrable intrinsic joint abnormality [28]. Although there are no specific ultrasound findings, findings associated with frozen shoulder syndrome may be correlated with increased fluid in the tendon sheath of the long head of the biceps [6]. Two reports have mentioned an association between chronic tendinopathy and the increase in thickness of the rotator cuff through indirect or direct measurements [24, 29]. Regarding the sonography findings, there is a significantly thicker bicipital effusion in PD patients with asymptomatic shoulders than in the shoulders of the controls. Furthermore, bicipital effusion is significantly thicker in PD patients with symptomatic shoulders than in those with asymptomatic shoulders.

A thicker mean bicipital effusion is observed in patients with significantly higher mean ipsilateral UPDRS III and its subscores for tremor, rigidity, and bradykinesia (scores indicated in advanced PD). This study also demonstrates the effects of ipsilateral UPDRS III and its subscores on the thickness of the supraspinatus and subscapularis tendons and the significantly higher tendon thickness of both the supraspinatus and the subscapularis in asymptomatic PD shoulders compared to those of the controls. The supraspinatus tendon is considered to be the most commonly affected site by degeneration and among rotator cuff age-related increases in thickness [24]. The present study demonstrates that not only the supraspinatus tendon but also the subscapularis tendon has changes associated with the clinical severity of PD, which is represented by ipsilateral UPDRS III and its subscores.

One study indicates that the supraspinatus and subscapularis tendons are especially commonly affected and cause an anterosuperior subluxation of the shoulder joint in patients with PD [30]. Another study shows positive correlations between supraspinatus tendon tear and ipsilateral tremor and rigidity scores [9]. Consistent with such findings, results of the present study also reveal a positive correlation between the thickness of the supraspinatus tendon and ipsilateral UPDRS III and its subscores for tremor, rigidity, and bradykinesia. The mean infraspinatus tendon thickness is the only parameter that does not show any significant correlation with clinical motor scores because the thickness of the infraspinatus tendon is relatively difficult to measure due to the lack of anatomic landmarks.

Several articles report risk factors for frozen shoulder syndrome, including prolonged illness, rigidity, immobilization, and akinesia [7, 8, 31]. One study demonstrates that akinesia is common as a first manifestation and is correlated with the laterality of the frozen shoulder [7]. Another study demonstrates that rigidity and bradykinesia in PD patients are supposed to lead to limited joint range of motion and predispose the patient to developing frozen shoulder syndrome [7, 31]. The present study demonstrated the relationship between clinical severity and thickness of bicipital effusion. Patients with lower mean MMSE score, which may imply cognitive decline, have thicker bicipital effusion. Several important risk factors for frozen shoulders (e.g., age, sex, occupation, disease duration, and PD phenotypes) should be further discussed. In the present study, lower educational level and disease duration were found in patients without frozen shoulder. However the difference is not significant within our patients. Our study is limited to small patients' number and patients with lower educational level, and thus the occupation and PD phenotypes difference were not studied in the present study.

Although this study demonstrates that the mean ipsilateral tremor score in UPDRS III is independently associated with frozen shoulder syndrome in patients with idiopathic PD, it has several limitations. First, PD is a slowly progressive neurodegenerative disorder. Hence, it is not possible to assess the effects of disease progression on the duration and severity of frozen shoulder in a cross-sectional study. Second, as a prospective observation study, it may be subject to bias of unmeasured factors. The frozen shoulders of some patients may be related to accidents or immobilization. Furthermore, underlying cognitive decline may cause potential frozen shoulder during follow-up. Third, PD patients with frozen shoulder syndrome associated with rotator cuff tear have been excluded and this may be subject to bias in statistical analysis. Lastly, the case numbers are small. Large-scale prospective studies to clarify the relationship are warranted.

Considering the morbidity and mortality associated with frozen shoulder syndrome, prevention and evaluation of shoulder through sonography should be considered in high-risk patients with PD, especially in those with higher UPDRS III. These are important issues for patient safety and are highly relevant for improving their quality of life.

## Figures and Tables

**Table 1:** Demographic data of patients with Parkinson's disease.

	PD (*n* = 30)
Age	64.77 ± 10.3
Male (*n*, %)	14, 46.7%
Disease duration, years	5.07 ± 3.2
Hoehn and Yahr staging	2.12 ± 1.0
Unified Parkinson's Disease Rating Scale (UPDRS) III	14.77 ± 8.4
Tremor score	3.63 ± 3.6
Rigidity score	4.47 ± 3.1
Bradykinesia score	7.87 ± 4.2
Levodopa equivalent dose (mg)	411.48 ± 344.3
Mini-Mental State Examination	24.57 ± 6.1
Clinical Dementia Rating	
0 (*n*, %)	16, 53.3%
0.5 (*n*, %)	13, 43.3%
1 (*n*, %)	1, 3.3%
Underlying conditions	
Hypertension (*n*, %)	12, 40.0%
Diabetes mellitus (*n*, %)	6, 20%
Knee osteoarthritis	4, 13%

Values are expressed in mean ± SD unless otherwise indicated.

**Table 2:** Clinical data and shoulder sonography findings among healthy controls and PD patients with and without frozen shoulder syndrome.

	Control (*N* = 17)	PD (*N* = 30)		*p* value
		With frozen shoulder (*n* = 41)	Without frozen shoulder (*n* = 19)	
Age	63.71 ± 9.6	64.77 ± 10.3		
Sex (male/female)	6/11	14/16		
Tendon thickness (mm)				
Subscapularis	4.38 ± 1.0	4.95 ± 1.1	5.10 ± 1.2	0.630^a^
Supraspinatus	4.42 ± 1.0	5.06 ± 1.1	5.45 ± 1.3	0.184^a^
Infraspinatus	3.58 ± 0.6	3.80 ± 0.7	3.24 ± 0.4	<0.001^a^
Thickness of bicipital effusion (mm)	1.39 ± 0.3	2.08 ± 0.8	2.72 ± 0.5	0.002^a^
Ipsilateral motor				
Ipsilateral UPDRS III		14.07 ± 8.8	17.00 ± 7.1	0.210^b^
Ipsilateral tremor score (median [IQR])		1 (0, 2)	2 (0.5, 3.5)	0.065^b^
Ipsilateral rigidity score		1.98 ± 1.7	2.79 ± 1.6	0.083^b^
Ipsilateral bradykinesia score		3.73 ± 2.4	4.37 ± 2.1	0.333^b^
Contralateral TRODAT ratio		1.49 ± 0.1	1.28 ± 0.1	<0.001^b^

Values are expressed in mean ± SD unless otherwise indicated.

^a^Statistical comparison by ANOVA.

^b^Statistical comparison by independent *t*-test.

PD, Parkinson's disease; IQR, interquartile range; MMSE, Mini-Mental State Examination; UPDRS, Unified Parkinson's Disease Rating Scale.

**Table 3:** Correlation between parameters of shoulder sonography findings and clinical severity in PD patients.

Variable	Thickness of bicipital effusion	Subscapularis thickness	Supraspinatus thickness	Infraspinatus thickness
Age, years	0.175	0.138	0.254	−0.057
Body mass index, kg/m^2^	0.124	0.115	0.145	−0.094
Disease duration, years	0.105	0.143	0.166	−0.102
Mini-Mental State Examination	−0.341^*∗*^	−0.123	−0.028	0.030
Striatal dopamine transporter uptake ratios				
Contralateral TRODAT ratio	−0.319	−0.081	−0.367^*∗*^	0.122
Ipsilateral TRODAT ratio	0.030	0.139	−0.100	0.105
Levodopa equivalent dose (mg)	0.027	−0.139	−0.003	−0.092
Hoehn and Yahr staging	0.321^*∗*^	0.162	0.055	0.289^*∗*^
Clinical motor state				
Ipsilateral motor state				
Ipsilateral UPDRS III	0.618^*∗∗*^	0.386^*∗*^	0.421^*∗∗*^	0.110
Ipsilateral tremor score	0.521^*∗∗*^	0.456^*∗∗*^	0.341^*∗*^	−0.073
Ipsilateral rigidity score	0.574^*∗∗*^	0.277^*∗*^	0.207	−0.132
Ipsilateral bradykinesia score	0.613^*∗∗*^	0.400^*∗*^	0.385^*∗*^	0.135
Contralateral motor state				
Contralateral UPDRS III	0.255	0.089	0.225	0.066
Contralateral tremor score	0.095	0.140	0.164	0.120
Contralateral rigidity score	0.222	0.022	0.035	0.047
Contralateral bradykinesia score	0.169	0.044	0.174	0.011

Values indicate correlation coefficient.

^*∗*^ *p* < 0.05; ^*∗∗*^ *p* < 0.01.

PD, Parkinson's disease; UPDRS, Unified Parkinson's Disease Rating Scale.
